# Mitochondria-Targeted Colorimetric and Ratiometric Fluorescent Probe for Hg^2+^ with Large Stokes Shift

**DOI:** 10.3390/molecules31122092

**Published:** 2026-06-14

**Authors:** Dongjian Zhu, Yufei Zhang, Yuyan Pan, Sheng Li, Aishan Ren

**Affiliations:** Guangxi Key Laboratory of Health Care Food Science and Technology, College of Food and Bioengineering, Hezhou University, Hezhou 542899, China; dongjianzhu22118@163.com (D.Z.); ljian0614@163.com (Y.Z.); pyy19950116@163.com (Y.P.); 18741087925@163.com (S.L.)

**Keywords:** mitochondria-targeted, colorimetric and ratiometric, fluorescent probe, Hg^2+^, large stokes shift

## Abstract

In this study, probe **1**, a novel mitochondria-targeted fluorescent probe for the colorimetric and ratiometric detection of Hg^2+^, was developed. Upon addition of Hg^2+^ to the solution of **1**, distinct spectral changes were observed. The absorption spectra underwent a blue shift from 510 nm to 450 nm (Δλ = 60 nm), and the solution color changed from red to pale yellow under daylight. Concurrently, a significant blue shift occurred from 645 nm to 540 nm (Δλ = 105 nm) in the fluorescence spectra. There were remarkable variations in the fluorescence intensity ratio of F_540 nm_/F_645 nm_ with the R/R_0_ value reaching up to 824-fold, and the fluorescence color changed from red to green under a 365 nm UV lamp. Probe **1** featured a large Stokes shift of 135 nm, high sensitivity with an LOD of 25.5 nm, and excellent selectivity for Hg^2+^ even in the presence of other analytes. Furthermore, **1** was successfully applied for the ratiometric imaging of intracellular Hg^2+^ and was confirmed to localize specifically within mitochondria.

## 1. Introduction

Heavy metal contamination poses a serious global environmental threat. Mercury, in particular, is among the most toxic heavy metals and commonly exists in inorganic, organic, and elemental forms [1]. Among these, the mercury ion (Hg^2+^) represents the predominant form occurring in natural environments. Mercury pollution arises from both natural sources like volcanic activity and anthropogenic activities including mining operations, chemical production, combustion of fossil fuels, and discharges of industrial and municipal wastewater [2]. Widespread release of mercury into air, water, and soil facilitates its incorporation into the food chain, eventually leading to human exposure [3]. Due to its non-biodegradable nature, mercury readily accumulates in the human body. It exhibits high affinity for sulfur- and selenium-containing residues in proteins and enzymes, resulting in disruption of biological functions [4]. Such interactions contribute to severe health impairments, including kidney damage, Minamata disease, neurological and cognitive disorders, central nervous system injuries, and increased cancer risk [5,6]. Therefore, developing effective methods for Hg^2+^ detection is crucial for environmental monitoring and safeguarding public health.

To date, a diverse array of conventional analytical techniques has been established for Hg^2+^ detection, encompassing electrochemical analysis [7], high-performance liquid chromatography (HPLC) [8], atomic fluorescence/absorption spectrometry (AFS/AAS) [9,10], inductively coupled plasma–optical emission spectrometry/mass spectrometry/atomic emission spectrometry (ICP-OES/ICP-MS/ICP-AES) [11,12,13], surface plasmon resonance (SPR) [14], surface-enhanced Raman scattering (SERS) [15], X-ray absorption spectroscopy (XAS) [16], and the colorimetric method [17]. Nevertheless, these traditional methods are constrained by inherent limitations that hinder their applicability for rapid, real-time, and in situ Hg^2+^ detection in living systems. Specifically, they often demand expensive equipment, involve tedious operation procedures, require sophisticated sample preparation, consume significant time, and necessitate professional personnel. In contrast, the fluorescent probe has emerged as a highly promising alternative for Hg^2+^ detection in biological systems. Its superiority stems from inherent advantages of low cost, easy synthesis, high sensitivity and selectivity, ease of operation, low sample consumption, rapid response, non-invasiveness, and real-time imaging capabilities with high spatiotemporal resolution [18,19]. These attributes collectively make the fluorescent probe well-suited for bridging the gap left by conventional techniques in biological Hg^2+^ detection scenarios.

Consequently, the development of fluorescent probes has attracted significant research attention, leading to the construction of numerous fluorescent probes for Hg^2+^ detection [20,21,22,23,24,25,26,27,28,29,30,31,32,33,34,35,36,37,38,39,40,41]. Coordination, a common approach in designing Hg^2+^ fluorescent probes, has several drawbacks. It often results in fluorescence quenching due to the spin orbit coupling effect. Moreover, competitive coordination with other metal ions can generate false-positive signals [23]. In contrast, reaction-based fluorescent probes have garnered more interest owing to their superior selectivity toward Hg^2+^ [24,25,26,27,28,29,30,31,32,33,34,35,36,37,38,39,40,41]. These reaction-based fluorescent probes for Hg^2+^ can be classified into five main categories according to their recognition groups. The first category includes sulfur-containing recognition groups, such as thione [24], thioamide [25], thioimide [26], thiourea [27], carbonothioate [28], thiocarbamate [29], thiophosphate [30], dithioacetal [31], thioether [32], and thiorhodamine [33]. The second category consists of selenium-containing recognition groups, for example, selenolactone [34,35], diphenylphosphinoselenoate [36,37], selone [4], and phosphane selenide [38]. Alkyne-containing recognition groups [39], vinyl ether-containing recognition groups [40], and boronic acid-containing recognition groups [41] form the remaining three categories. Although sulfur-containing recognition motifs have been widely employed in the design of Hg^2+^ fluorescent probes, selenium-based fluorescent probes exhibit greater potential for enhanced selectivity and sensitivity. This advantage stems from two key factors: First, selenium is an indispensable trace element in living systems and acts as a well-accepted biological antagonism to Hg^2+^. Notably, the binding affinity of Hg^2+^ for selenium is approximately six orders of magnitude higher than that for sulfur, driven by a strong antagonistic interaction [36]. Second, the solubility of HgSe is at least ten orders of magnitude lower than that of HgS [42]. Consequently, several selenium-based Hg^2+^ fluorescent probes employing fluorophores such as fluorescein [36], rhodamine [34,35], heptamethine cyanine [4], coumarin [37], and polyphenylethynyl [38] have been reported. However, most of these selenium-based fluorescent probes operate via turn-on fluorescence at a single emission wavelength, which is susceptible to environmental factors and instrumental conditions [34,35,36,38]. In contrast, ratiometric fluorescent probes, which rely on the ratio of emission intensities at two different wavelengths for self-correction, can mitigate most of these interferences [4,27,28,30,37,43,44], thereby granting higher sensitivity and measurement precision. Nevertheless, selenium-based ratiometric fluorescent probes for Hg^2+^ detection remain scarce [4,37]. Some researchers have indicated that Hg^2+^ has a tendency to accumulate in mitochondria, which can lead to a decrease in mitochondrial membrane potential, induce oxidative stress, and even trigger cell apoptosis [45]. Consequently, the development of mitochondria-targeted Hg^2+^ fluorescent probes is of particular importance, yet only one selenium-based mitochondria-targeted Hg^2+^ fluorescent probe has been reported by our group to date [37]. Additionally, integrating colorimetric detection further enhances practical value due to its simplicity, cost-effectiveness, and instrument portability [46]. Recently, our group pioneered the derivation of the diphenylphosphinoselenoic group into a benzyl bromide derivative and designed the first coumarin-based mitochondria-targeted ratiometric and colorimetric fluorescent probe for Hg^2+^ detection [37]. In this probe, a pyridine moiety was incorporated at the 3-position of 7-diethylaminocoumarin via a carbon–carbon single bond (C-C), but it exhibited a relatively small Stokes shift (<100 nm). As is well-established, fluorescent probes with large Stokes shifts are highly desirable, as they minimize self-absorption and enhance signal accuracy [47]. Therefore, the design of mitochondria-targeted colorimetric and ratiometric Hg^2+^ fluorescent probes with large Stokes shift remains highly desirable yet challenging task.

**Scheme 1:**
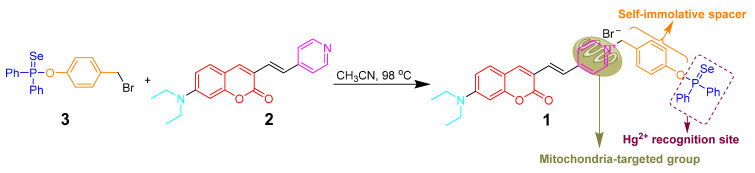
Synthetic strategy for **1**.

In our preceding work, we engineered a coumarin-derived fluorophore **2** (Scheme 1) by incorporating a pyridine unit at the 3-position of 7-diethylaminocoumarin via a carbon–carbon double bond (C=C). This structural design endows the molecule with a distinct donor–π–acceptor (D-π-A) configuration: the diethylamino group functions as the electron donor, while the pyridine ring acts as the electron acceptor. This molecular design yielded excellent photophysical properties, including a large Stokes shift and high fluorescence quantum yield [47]. Moreover, the electron-withdrawing capacity of the acceptor can be finely adjusted through structural interconversion between pyridine and pyridinium, rendering this scaffold a promising platform for constructing colorimetric and ratiometric fluorescent probes. Building upon this foundation, we herein designed and synthesized a mitochondria-targeted colorimetric and ratiometric fluorescent probe (*E*)-4-(2-(7-(diethylamino)-2-oxo-2*H*-chromen-3-yl)vinyl)-1-(4-((diphenylphosphoroselenoyl)oxy)benzyl)pyridin-1-ium bromide (termed **1**) for the detection of Hg^2+^, which integrates coumarin as the fluorophore, a diphenylphosphinoselenoic group as the recognition site, and a *p*-hydroxybenzyl moiety as the self-immolative spacer (Scheme 1). As expected, **1** exhibited colorimetric and ratiometric fluorescence responses toward Hg^2+^, characterized by high sensitivity, excellent selectivity, and a large Stokes shift. Moreover, **1** was successfully applied for ratiometric imaging of Hg^2+^ in HeLa cells and demonstrated mitochondrial targeting capability attributed to the positive charge of the pyridinium unit.

## 2. Results

### 2.1. Response of ***1*** Toward Hg^2+^ and Proposed Sensing Mechanism

**Figure 1:**
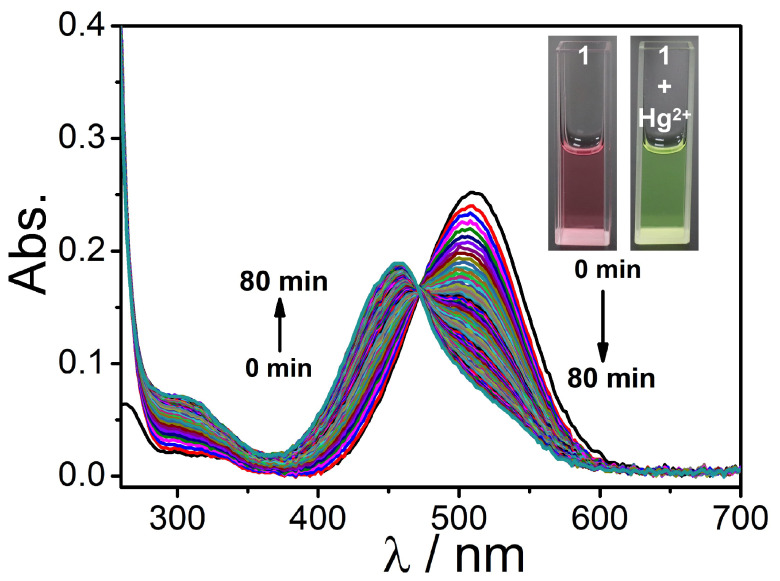
Absorption spectra of **1** (5 μM) after addition of Hg^2+^ (150 μM) in 10 mM PBS buffer solution/DMSO (1:1, *v*/*v*, pH = 7.4, 25 °C). Insets: The color of **1** before and after reaction with Hg^2+^ (150 μM) under daylight.

**Figure 2:**
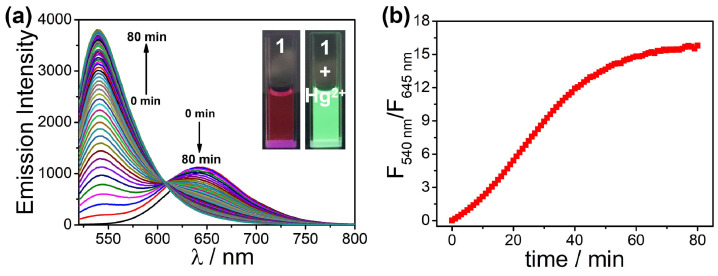
(**a**) Fluorescence spectra of **1** (5 μM) after addition of Hg^2+^ (150 μM). Insets: The fluorescence color of **1** before and after reaction with Hg^2+^ (150 μM) under a 365 nm UV lamp. (**b**) The fluorescence intensity ratio (F_540 nm_/F_645 nm_) changes as a function of time. Data were acquired in a 10 mM PBS buffer solution/DMSO (1:1, *v*/*v*, pH = 7.4, 25 °C, λ_ex_ = 500 nm).

**Scheme 2:**
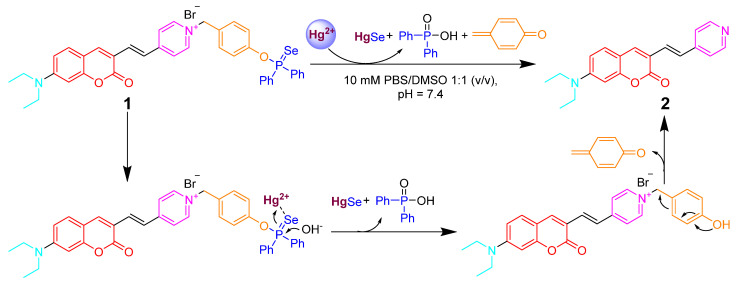
Proposed sensing mechanism of **1** for Hg^2+^.

The absorption and fluorescence responses of **1** toward Hg^2+^ were investigated in a 10 mM PBS buffer solution/DMSO (1:1, *v*/*v*, pH = 7.4, 25 °C). For absorption spectra, **1** (5 μM) exhibited a maximum absorption peak at 510 nm with a molar extinction coefficient (ε_max_) of 5.40 × 10^4^ M^−1^ cm^−1^ (Figure 1). Upon addition of Hg^2+^ (150 μM), the absorbance at 510 nm decreased progressively, while a new blue-shifted absorption peak at 450 nm increased steadily, accompanied by a distinct isosbestic point at 472 nm (Figure 1). A distinct color change from red to pale yellow was observed under daylight (Figure 1 insets), enabling colorimetric detection of Hg^2+^ by the naked eye. Upon excitation at 500 nm, **1** (5 μM) displayed a maximum fluorescence emission peak at 645 nm (*Φ*_f_ ≈ 0.51 in DMSO) with a large Stokes shift of 135 nm (Figure 2a). Introduction of Hg^2+^ (150 μM) resulted in a gradual decrease in the fluorescence emission peak at 645 nm and a concurrent increase in the new blue-shifted fluorescence emission peak at 540 nm (Figure 2a). This dual fluorescence emission change was accompanied by a noticeable fluorescence color transition from red to green under a 365 nm UV lamp (Figure 2a insets). The fluorescence intensity ratio (F_540 nm_/F_645 nm_) increased dramatically from 0.018 to 14.832 (over 824-fold enhancement) and reached a stable plateau within 60 min (Figure 2b). These results confirmed that **1** could achieve sensitive, colorimetric, and ratiometric detection of Hg^2+^ utilizing the fluorescence intensity ratio between 540 nm and 645 nm (F_540 nm_/F_645 nm_). Notably, the absorption (Figure S1a) and fluorescence (Figure S1b) profiles of **1** after treatment with Hg^2+^ closely resembled those of **2** (ε_max_ = 4.42 × 10^4^ M^−1^ cm^−1^, *Φ*_f_ ≈ 0.77 in DMSO). The solution colors under both daylight (Figure S1a insets) and a 365 nm UV lamp (Figure S1b insets) were also consistent with those of **2**. Based on our previous studies [37], these behaviors were attributable to Hg^2+^-induced deselenation–hydrolysis of the diphenylphosphinoselenoate moiety in **1** with cleavage of the P-O bond and release of the hydroxyl group, with a subsequent self-immolative reaction of the *p*-hydroxybenzyl moiety by 1,6-elimination to produce **2** (Scheme 2). Further validation was provided by ESI-MS analysis, which showed an obvious peak at *m*/*z* = 321.1 [M + H]^+^, corresponding to **2** (Figure S2). These results collectively confirmed the proposed sensing mechanism of **1** for Hg^2+^ (Scheme 2).

### 2.2. Optimization of Experimental Conditions

We tested the effect of different volume ratios of DMSO on the detection of Hg^2+^ by **1** in a 10 mM PBS buffer solution (pH = 7.4, 25 °C). As displayed in Figure S3, a progressive increase in the fluorescence intensity ratio (F_540 nm_/F_645 nm_) was observed with the volume fraction of DMSO ranging from 0% to 50%. However, when the volume fraction of DMSO increased from 50% to 80%, the reaction between **1** and Hg^2+^ slowed down (Figure S3a) and the fluorescence intensity ratio (F_540 nm_/F_645 nm_) decreased (Figure S3b). Therefore, we chose the 10 mM PBS buffer solution/DMSO at a ratio of 1:1 (*v*/*v*). Next, the solubility of **1** and **2** were assessed in the 10 mM PBS buffer solution/DMSO (1:1, *v*/*v*, pH = 7.4, 25 °C) using fluorescence spectra. As illustrated in Figure S4, both **1** and **2** demonstrated satisfactory solubility under this condition. Furthermore, the pH dependence of the fluorescence behavior was investigated for **1** both in the absence and presence of Hg^2+^, as well as for **2** alone, by monitoring the fluorescence intensity ratio of F_540 nm_/F_645 nm_ (Figure S5). In the case of **1** (5 μM) without Hg^2+^ addition, as the pH was gradually increased from 2.0 to 10.0, the fluorescence intensity ratio (F_540 nm_/F_645 nm_) remained nearly constant (Figure S5), indicating **1** had high stability under various acidic–basic conditions. However, when Hg^2+^ (150 μM) was present, the fluorescence intensity ratio (F_540 nm_/F_645 nm_) of **1** exhibited distinct pH-dependent behavior. It remained unchanged in the pH range of 2.0–6.0, then increased sharply between pH 6.0 and 8.0, and finally leveled off again in the pH range of 8.0–10.0 (Figure S5b). Remarkably, **2** (5 μM) showed different pH-induced fluorescence changes. The fluorescence intensity ratio (F_540 nm_/F_645 nm_) of **2** increased significantly as the pH was adjusted from 2.0 to 6.0 and then remained stable in the pH range of 6.0–10.0 (Figure S5a). At pH 2.0, **2** displayed a maximum fluorescence emission peak at 645 nm (Figure S6a). However, upon increasing the pH from 3.0 to 10.0, this maximum fluorescence emission peak underwent a substantial blue shift from 645 nm to 540 nm (Figure S6a). This phenomenon can be attributed to the protonation of nitrogen (N) atoms of the pyridine under acidic conditions, which enhanced the intramolecular charge transfer (ICT) effect within **2**. By plotting the fluorescence intensity ratio (F_540 nm_/F_645 nm_) as a function of pH, the pK_a_ value of **2** was determined to be 3.81 ± 0.02 (Figure S6b). These findings suggest that **1** is suitable for Hg^2+^ sensing under physiological conditions. Therefore, the 10 mM PBS buffer solution/DMSO (1:1, *v*/*v*, pH = 7.4, 25 °C) was selected as the optimized condition for subsequent experiments.

### 2.3. Quantitative Assay of Hg^2+^ by ***1***

**Figure 3:**
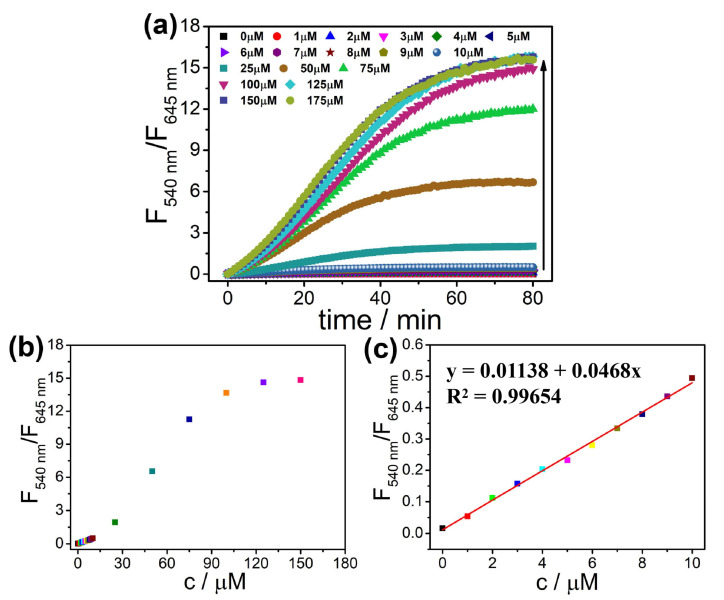
(**a**) Plot of fluorescence intensity ratio of F_540 nm_/F_645 nm_ versus the reaction time after addition of various concentrations of Hg^2+^ (from bottom to top): 0 (control), 1, 2, 3, 4, 5, 6, 7, 8, 9, 10, 25, 50, 75, 100, 125, 150 and 175 µM. (**b**) The fluorescence intensity ratio (F_540 nm_/F_645 nm_) of **1** as a function of Hg^2+^ concentration. (**c**) The linear relationship between the fluorescence intensity ratio (F_540 nm_/F_645 nm_) of **1** and Hg^2+^ concentration in the range of 0–10 μM. Data were acquired in the 10 mM PBS buffer solution/DMSO (1:1, *v*/*v*, pH = 7.4, 25 °C, λ_ex_ = 500 nm).

To evaluate the quantitative detection performance of **1** for Hg^2+^, fluorescence titration experiments were conducted. Probe **1** was maintained at a constant concentration of 5 µM, while the concentration of Hg^2+^ was gradually increased from 0 to 175 µM. As shown in Figure 3a, the fluorescence intensity ratio (F_540 nm_/F_645 nm_) of **1** alone remained consistently low, which further confirmed the good stability of **1** in the detection system. Upon incremental addition of Hg^2+^ (0–150 µM), the fluorescence intensity ratio (F_540 nm_/F_645 nm_) of **1** increased progressively and eventually reached a saturation plateau (Figure 3b). A good linear relationship (*y* = 0.01138 + 0.0468*x*, R^2^ = 0.99654) was observed between the fluorescence intensity ratio (F_540 nm_/F_645 nm_) and Hg^2+^ concentration in the range of 0 to 10 µM (Figure 3c). The LOD of **1** for Hg^2+^ was determined to be 25.5 nM, which was similar to the reported selenium-based fluorescent probes (Table S1). These results proved that **1** could realize quantitative and sensitive detection of Hg^2+^.

### 2.4. The Selectivity of ***1*** for Hg^2+^

**Figure 4:**
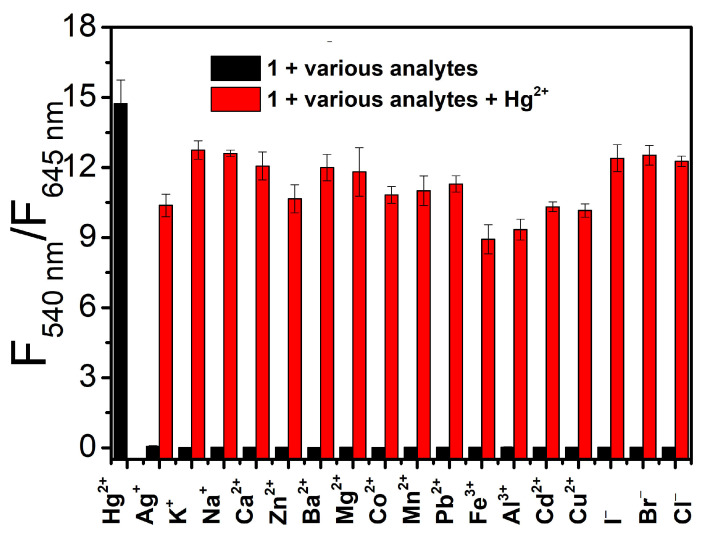
The ratios of fluorescence intensities (F_540 nm_/F_645 nm_) of **1** (5 μM) after the addition of various analytes (150 µM) in the 10 mM PBS buffer solution/DMSO (1:1, *v*/*v*, pH = 7.4, 25 °C, λ_ex_ = 500 nm).

To evaluate the selectivity of **1**, its absorption and fluorescence responses toward various analytes (150 μM) were investigated. As shown in Figure S7a, only Hg^2+^ induced a pronounced blue shift in the absorption peak of **1** from 510 nm to 450 nm, whereas other analytes, such as Ag^+^, K^+^, Na^+^, Ca^2+^, Zn^2+^, Ba^2+^, Mg^2+^, Co^2+^, Mn^2+^, Pb^2+^, Fe^3+^, Al^3+^, Cd^2+^, Cu^2+^, I^−^, Br^−^ and Cl^−^, caused only minor spectral changes. The anti-interference ability was further assessed by measuring the absorption response of **1** to Hg^2+^ in the presence of other analytes. As illustrated in Figure S7b, the characteristic absorption peak at 450 nm remained clearly discernible without significant interference, confirming excellent specificity for Hg^2+^. Fluorescence responses were consistent with the absorption results; a remarkable increase in the fluorescence intensity ratio (F_540 nm_/F_645 nm_) was observed upon addition of Hg^2+^, either alone or in combination with other analytes (Figure 4), accompanied by a shift in the maximum fluorescence emission peak to 540 nm (Figure S8). In contrast, the presence of other analytes alone resulted in negligible changes in the fluorescence intensity ratio (F_540 nm_/F_645 nm_) (Figure 4), with the maximum fluorescence emission peak remaining at 645 nm (Figure S8a).

**Figure 5:**
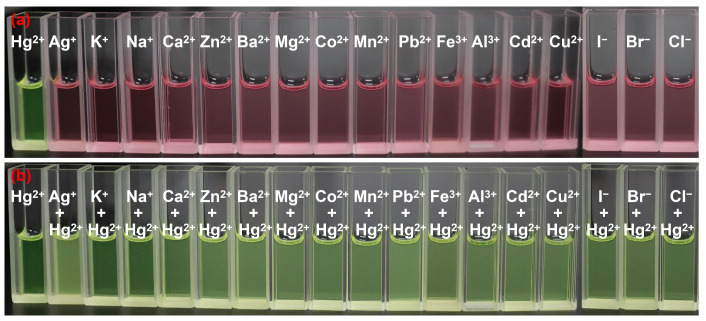
The color of **1** (5 µM) after addition of various analytes (150 μM) in the 10 mM PBS buffer solution/DMSO (1:1, *v*/*v*, pH = 7.4, 25 °C) under daylight: (**a**) **1** treated with the marked analytes; (**b**) **1** treated with the marked analytes followed by Hg^2+^.

**Figure 6:**
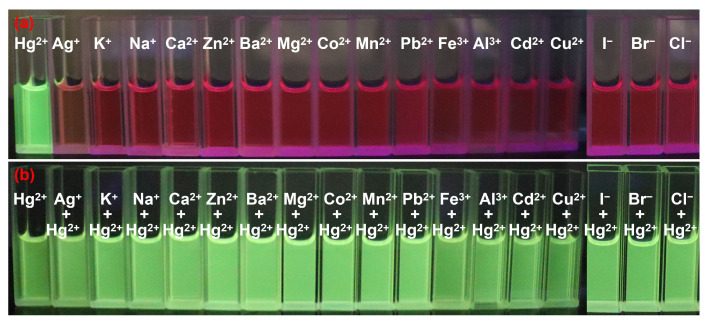
The fluorescence color of **1** (5 µM) after addition of various analytes (150 μM) in the 10 mM PBS buffer solution/DMSO (1:1, *v*/*v*, pH = 7.4, 25 °C) under a 365 nm UV lamp: (**a**) **1** treated with the marked analytes; (**b**) **1** treated with the marked analytes followed by Hg^2+^.

Additionally, **1** enabled visual detection of Hg^2+^ through distinct color changes: the solution turned pale yellow under daylight (Figure 5) and green under a 365 nm UV lamp (Figure 6). These changes were consistently observed both in the absence and presence of other analytes. In contrast, the solution containing other analytes exhibited red color under both daylight (Figure 5a) and a 365 nm UV lamp (Figure 6a). Collectively, these results demonstrated that **1** possessed outstanding selectivity and anti-interference performance toward Hg^2+^, making it a promising probe for reliable Hg^2+^ detection in complex environments.

### 2.5. Cell Imaging

**Figure 7:**
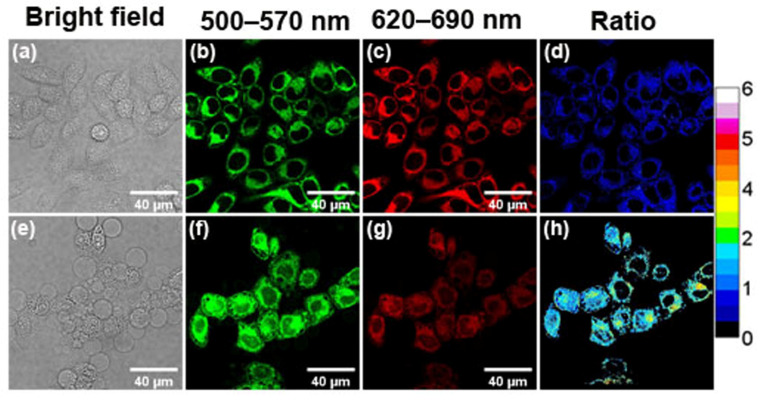
Fluorescence imaging of Hg^2+^ in HeLa cells with **1**. (**a**–**d**) Cells were treated with **1** (5 μM) at 37 °C for 30 min. (**e**–**h**) Cells were successively treated with **1** (5 μM) for 30 min and Hg^2+^ (200 μM) for 30 min at 37 °C. From left to right: bright field, Em_500–570 nm_, Em_620–690 nm_, ratio images of Em_500–570 nm_/Em_620–690 nm_. Scale bar: 40 μm. Ratio bar: 0–6. λ_ex_ = 488 nm.

The promising in vitro performance of **1** prompted us to explore its applicability for imaging Hg^2+^ in living cells. First, the cytotoxicity of **1** toward HeLa cells was evaluated using the CCK-8 assay. As shown in Figure S9, even at a concentration of 10 μM, **1** exhibited low cytotoxicity with cell viability exceeding 94%. Subsequently, we applied **1** for the ratiometric fluorescence imaging of Hg^2+^ in HeLa cells (Figure 7). The fluorescence signals were acquired in two emission channels: green (500–570 nm) and red (620–690 nm). HeLa cells incubated with **1** (5 μM, 30 min, 37 °C) exhibited detectable fluorescence signals in both the green (Figure 7b) and red channels (Figure 7c) and gave an average ratio value (Em_500–570 nm_/Em_620–690 nm_) of 0.98 (Figure 7d), indicating that **1** could readily permeate the cell membrane. When HeLa cells were first treated with **1** (5 μM, 30 min, 37 °C) and then treated with Hg^2+^ (200 μM, 30 min, 37 °C), an increase in the green channel signal (Figure 7f) and a decrease in the red channel signal (Figure 7g) were observed; meanwhile, enhancements in the ratio were obtained with an average value of 1.36 (Figure 7h), which can be attributed to the increase in intracellular Hg^2+^. These results confirmed that **1** can serve as a reliable ratiometric fluorescent probe for visualizing Hg^2+^ in living cells.

**Figure 8:**
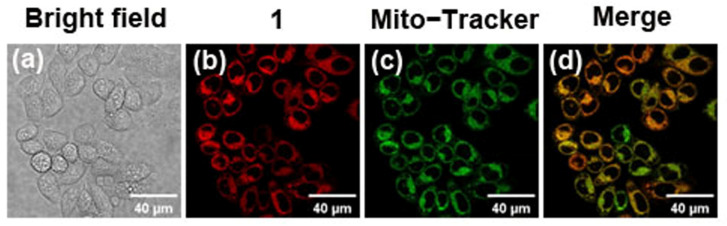
Fluorescence imaging of HeLa cells co-stained with **1** (5 μM) and Mito-Tracker Green (500 nM) for 30 min at 37 °C. (**a**) Bright-field image; (**b**) emission intensities were collected in an optical window of 620–690 nm for **1**; (**c**) emission intensities were collected in an optical window of 495–530 nm for Mito-Tracker Green; (**d**) overlay image of (**b**,**c**). Scale bar: 40 μm. λ_ex_ = 488 nm.

Given that **1** contains a pyridinium moiety bearing a positive charge, which conferred mitochondria-targeting ability and had been previously reported by our group [37,47], we performed colocalization experiments in HeLa cells. HeLa cells were co-stained with **1** (5 μM) and Mito-Tracker Green (500 nM, a well-established mitochondria-specific staining dye). When excited at 488 nm, **1** emitted fluorescence in the red channel (Figure 8b), while Mito-Tracker Green was detected in the green channel (Figure 8c). The satisfactory overlap between the two signals (Figure 8d) was quantitatively confirmed by a Pearson’s colocalization coefficient of 0.81, which was comparable to the reported pyridinium-based mitochondria-targeted fluorescent probes [48,49], indicating that **1** could effectively target in mitochondria.

## 3. Experimental Section

### 3.1. Reagents and Apparatus

Nuclear magnetic resonance spectra, including ^1^H NMR, ^13^C NMR and ^31^P NMR, were acquired using a Bruker (Billerica, MA, USA) Avance-400 NMR spectrometer operating at 400 MHz. Electrospray ionization mass spectra (ESI-MS) were recorded on an Agilent (Santa Clara, CA, USA) LC-MS 6120 system. pH values were determined with a FE20 pH meter. Confocal fluorescence imaging experiments were performed on a Leica (Wetzlar, Germany) STELLARIS STED microscope. Ultraviolet–visible (UV–vis) absorption spectra were collected with a Thermo Fisher Evolution 300 UV–vis spectrometer (Waltham, MA, USA). Fluorescence spectra were measured using a HITACHI F-4600 spectrometer (Marunouchi, Japan), where both the excitation and emission slit widths were fixed at 5 nm. Stock solutions of **1** and **2** (2 mM) were prepared by dissolving the respective solids in dimethyl sulfoxide (DMSO, spectrum grade). All aqueous solutions were prepared with ultrapure water (18.25 mΩ·cm). The solutions of analytes (10 mM) were prepared from the following salts: HgCl_2_, AgNO_3_, KCl, NaCl, CaCl_2_, ZnCl_2_, BaCl_2_·2H_2_O, MgCl_2_·6H_2_O, CoCl_2_·6H_2_O, MnSO_4_·H_2_O, PbCl_2_, FeCl_3_·6H_2_O, Al(NO)_3_·6H_2_O, CdCl_2_·2.5H_2_O, CuCl_2_·2H_2_O, NaBr and NaI. All the analytes were analytical grade with purity ≥98%. Phosphate-buffered saline (PBS) buffer solutions (10 mM) were prepared in ultrapure water.

### 3.2. Methods of Cell Culture

HeLa cells were sourced from the National Collection of Authenticated Cell Cultures (Shanghai, China). Dulbecco’s Modified Eagle Medium (DMEM), phosphate-buffered saline (PBS), and fetal calf serum (FCS) were procured from Beijing Solarbio Science & Technology Co., Ltd. (Beijing, China), while a streptomycin/penicillin solution (Hyclone) was supplied by Beyotime Biotechnology (Shanghai, China). HeLa cells were maintained in DMEM containing 10% FCS (Gibco, Grand Island, NY, USA) and 1% streptomycin/penicillin at 37 °C under a humidified atmosphere of 5% CO_2_. For imaging experiments, HeLa cells were seeded in 35 mM glass-bottom dishes and allowed to adhere for 48 h prior to staining. One group: HeLa cells were washed with PBS and bathed in serum-free DMEM (2 mL) with only **1** (5 μM) for 30 min at 37 °C, rinsed with PBS three times and bathed in PBS (1 mL) before imaging. The other group: HeLa cells were washed with PBS and bathed in serum-free DMEM (2 mL) with **1** (5 μM) for 30 min at 37 °C, and then the cells were washed with PBS and bathed in serum-free DMEM (2 mL) with 200 μM Hg^2+^ for 30 min at 37 °C, rinsed with PBS three times and bathed in PBS (1 mL) before imaging. For colocalization experiments, HeLa cells were washed with PBS and co-stained with **1** (5 μM) and Mito-Tracker Green (500 nM) in serum-free DMEM (2 mL) for 30 min at 37 °C, rinsed with PBS three times and bathed in PBS (1 mL) before imaging. Confocal fluorescence imaging experiments were performed on a Leica STELLARIS STED microscope with a 40× objective. The excitation wavelength of the laser was 488 nm.

### 3.3. Cytotoxicity Assay

HeLa cells were seeded into 96-well plates and pre-incubated for 24 h in a humidified incubator at 37 °C with a 5% CO_2_ atmosphere. Subsequently, the cells were treated with **1** at six gradient concentrations (0, 2, 4, 6, 8, and 10 μM) and further incubated for 12 h. After removing the culture medium containing **1**, 100 μL of Cell Counting Kit-8 (CCK-8) reagent was added to each well and incubated for 0.5 h. Finally, the absorbance value at 450 nm was measured using a microplate reader to evaluate cell viability.

### 3.4. Determination of the Detection Limit

The detection limit (LOD) was calculated on the basis of fluorescence titration data. First, a linear regression curve was constructed by plotting the fluorescence intensity ratio (F_540 nm_/F_645 nm_) against the Hg^2+^ concentration, and the slope (k) of this linear fitting curve was extracted. Subsequently, the fluorescence spectra of **1** (5 μM) in the 10 mM PBS buffer solution/DMSO (1:1, *v*/*v*, pH = 7.4, 25 °C, λ_ex_ = 500 nm) were collected 30 times, and the standard deviation (δ) of the blank signal was determined from these repeated measurements. Finally, the LOD was calculated using the following formula:

LOD = 3δ/k(1)

where δ represents the standard deviation of the blank measurements and k denotes the slope of the linear regression curve of the fluorescence intensity ratio (F_540 nm_/F_645 nm_) versus the Hg^2+^ concentration.

### 3.5. Determination of Quantum Yields

Fluorescence quantum yields (*Φ*) of **1** and **2** were determined using rhodamine B (*Φ*_f_ = 0.69 in anhydrous ethanol) [50] and fluorescein (*Φ*_f_ = 0.85 in 1 N NaOH) [51] as the references, respectively. The quantum yields of **1** and **2** were calculated using the following equation.

*Φ*_x_ = *Φ*_r_ (A_r_S_x_η_x_^2^)/(A_x_S_r_η_r_^2^) (2)

where *Φ* represents the fluorescence quantum yield, A denotes the absorbance at the selected excitation wavelength (ensuring A < 0.1 to avoid inner-filter effect), S is the integrated area under the corrected fluorescence emission spectrum, and η stands for the refractive index of the solvent used. The subscripts x and r refer to the unknown and the reference, respectively.

### 3.6. Synthesis of (E)-4-(2-(7-(Diethylamino)-2-oxo-2H-chromen-3-yl)vinyl)-1-(4-((diphenylphosphoroselenoyl)oxy)benzyl)pyridin-1-ium Bromide (***1***)

The synthetic strategy is illustrated in Scheme 1. Compounds **2** and **3** were synthesized according to our previously reported protocols [37,47]. The structure of compound **1** was fully characterized by ^1^H NMR, ^13^C NMR, ^31^P NMR and ESI-MS, which is provided in the Supporting Information.

Compound **2** [47] (160.2 mg, 0.50 mmol) and compound **3** [37] (337.7 mg, 0.75 mmol) were dissolved in acetonitrile (CH_3_CN) (8 mL) under an argon (Ar) atmosphere. The reaction mixture was then heated to 98 °C and stirred overnight. After completion of the reaction, the reaction mixture was cooled down to room temperature and further precipitated by adding ethyl acetate (EA). The crude product was collected by vacuum filtration and washed with EA. The precipitated solid was purified by silica gel column chromatography using dichloromethane (DCM)/methanol (MeOH) (50:1–20:1 *v*/*v*) as the eluent to afford desired product (**1**). Yield: 308.2 mg (80%). ^1^H NMR (400 MHz, DMSO-*d*_6_, ppm) *δ* 8.98 (d, *J* = 6.4 Hz, 2H), 8.24 (s, 1H), 8.17 (d, *J* = 6.8 Hz, 2H), 7.98–7.93 (m, 4H), 7.84 (d, *J* = 16.0 Hz, 1H), 7.67–7.56 (m, 7H), 7.53–7.50 (m, 3H), 7.15 (d, *J* = 7.6 Hz, 2H), 6.79–6.76 (m, 1H), 6.57 (d, *J* = 1.6 Hz, 1H), 5.69 (s, 2H), 3.48–3.43 (m, 4H), 1.12 (t, *J* = 6.8 Hz, 6H). ^13^C NMR (100 MHz, DMSO-*d*_6_, ppm) *δ* 159.53, 156.33, 153.73, 151.99, 145.58, 143.92, 137.39, 134.03, 133.06, 132.78, 132.75, 131.35, 131.17, 131.05, 130.78, 130.02, 128.96, 128.83, 123.63, 122.46, 122.00, 121.95, 113.58, 110.03, 108.39, 96.19, 44.36, 12.36. ^31^P NMR (162 MHz, DMSO-*d*_6_, ppm) *δ* 86.34. ESI-MS for C_39_H_36_N_2_O_3_PSe^+^ ([M − Br]^+^): calcd: 691.2, found: 691.2.

## 4. Conclusions

In summary, we developed a novel mitochondria-targeted colorimetric and ratiometric fluorescent probe, probe **1**, for Hg^2+^ detection. Upon interaction with Hg^2+^, **1** exhibited remarkable changes in both spectral and visual properties: a blue shift in absorption from 510 nm to 450 nm, a blue shift in fluorescence emission from 645 nm to 540 nm, and distinct color transitions from red to pale yellow under daylight and from red to green under a 365 nm UV lamp. The sensing mechanism involves Hg^2+^-promoted deselenation–hydrolysis of the diphenylphosphinoselenoate group in **1**, leading to cleavage of the P-O bond and formation of the hydroxyl group-containing intermediate. Subsequently, the *p*-hydroxybenzyl spacer underwent a 1,6-elimination-driven self-immolative reaction, ultimately releasing **2**. This pathway was corroborated by absorption spectroscopy, fluorescence spectroscopy, colorimetric analysis and ESI-MS. Probe **1** demonstrated a large Stokes shift (135 nm), high sensitivity (LOD of 25.5 nM), and exceptional selectivity for Hg^2+^ over other analytes. Moreover, **1** was successfully applied for ratiometric imaging of intracellular Hg^2+^ and exhibited specific mitochondrial localization.

## Data Availability

All data and materials described in this work are available in this article or in the Supplementary Materials.

## Supplementary Materials

The following supporting information can be downloaded at https://www.mdpi.com/article/10.3390/molecules31122092/s1.
