# Supplementary material for: Mitochondria-Targeted Colorimetric and Ratiometric Fluorescent Probe for Hg2+ with Large Stokes Shift
Source: Molecules. 2026 Jun 14;31(12):2092. doi: 10.3390/molecules31122092 (PMC13305874; doi:10.3390/molecules31122092)
Supplement: Supplementary file 1 [file molecules-31-02092-s001.zip › molecules-4360247-supplementary.pdf]

Supporting Information for

Mitochondria-Targeted Colorimetric and Ratiometric Fluorescent Probe for Hg^{2+} with Large Stokes Shift

Dongjian Zhu, Yufei Zhang, Yuyan Pan, Sheng Li and Aishan Ren *

Guangxi Key Laboratory of Health Care Food Science and Technology, College of Food and Bioengineering, Hezhou University, Hezhou 542899, China

* Correspondence: *rash_yudi@163.com*

Table S1. Comparison of selenium-based fluorescent probes for Hg^{2+}.

PROBES	EMISSION WAVELENGTH	RESPONSE TYPE	REACTION TIME	DETECTION LIMIT	STOKES SHIFT	TARGETING ORGANELLE	REF.
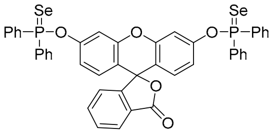	513 nm	Turn on	20 min	1.0 nM	21 nm	—	[S1]
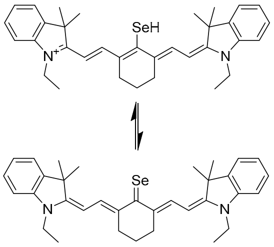	607 nm/800 nm<br>607 nm/567 nm	Ratiometric	30 s	—	45 nm<br>162 nm	—	[S2]
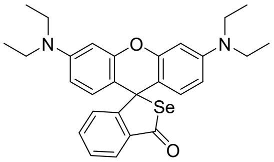	580 nm	Turn on	10 min	20 nM	20 nm	—	[S3]
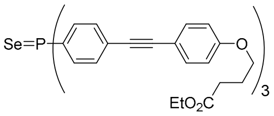	406 nm	Turn on	0.9 nM	0.9 nM	86 nm	—	[S4]
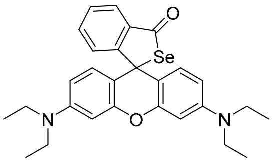	580 nm	Turn on	20 min	23 nM	20 nm	—	[S5]
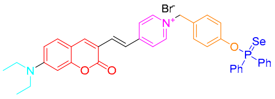	540 nm/645 nm	Ratiometric	60 min	25.5 nM	135 nm	Mitochondria	This work

References

[S1] Tang, B.; Ding, B.; Xu, K.; Tong, L. Use of Selenium to Detect Mercury in Water and Cells: An Enhancement of the Sensitivity and Specificity of a Seleno Fluorescent Probe. *Chem. Eur. J.* **2009**, 15, 3147-3151.

[S2] Wang, Y.; Gao, M.; Chen, Q.; Yu, F.; Jiang, G.; Chen, L. Associated Detection of Superoxide Anion and Mercury(II) under Chronic Mercury Exposure in Cells and Mice Models via a Three Channel Fluorescent Probe. *Anal. Chem.* **2018**, 90, 9769-9778.

[S3] Chen, X.; Baek, K.H.; Kim, Y.; Kim, S.J.; Shin, I.; Yoon, J. A selenolactone-based fluorescent chemodosimeter to monitor mecury/methylmercury species in vitro and in vivo. *Tetrahedron* **2010**, 66, 4016-4021.

[S4] Samb, I.; Bell, J.; Toullec, P.Y.; Michelet, V.; Leray, I. Fluorescent Phosphane Selenide As Efficient Mercury Chemodosimeter. *Org. Lett.* **2011**, 13, 1182-1185.
[S5] Shi, W.; Sun, S.; Li, X.; Ma, H. Imaging Different Interactions of Mercury and Silver with Live Cells by a Designed Fluorescence Probe Rhodamine B Selenolactone. *Inorg. Chem.* **2010**, 49, 1206-1210.

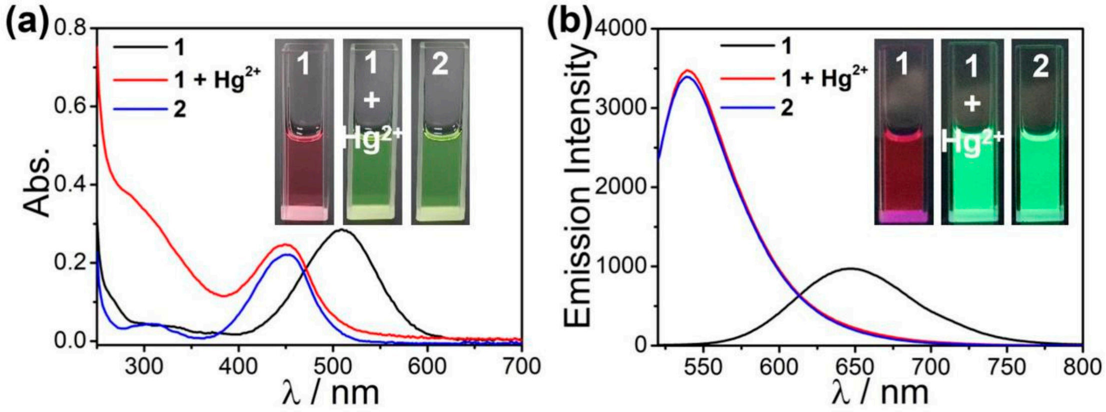

Figure S1. (a) Absorption spectra of **1** (5 μM) (black line) before and after reaction with Hg^{2+} (150 μM) (red line), as well as **2** (5 μM) (blue line) in 10 mM PBS buffer solution/DMSO (1:1, v/v, pH = 7.4, 25 °C). Insets: The color of **1** before and after reaction with Hg^{2+}, as well as **2** under daylight. (b) Fluorescence spectra of **1** (5 μM) (black line) before and after reaction with Hg^{2+} (150 μM) (red line), as well as **2** (5 μM) (blue line) in 10 mM PBS buffer solution/DMSO (1:1, v/v, pH = 7.4, 25 °C, λ_{ex} = 500 nm). Insets: The fluorescence color of **1** before and after reaction with Hg^{2+}, as well as **2** under a 365 nm UV lamp.

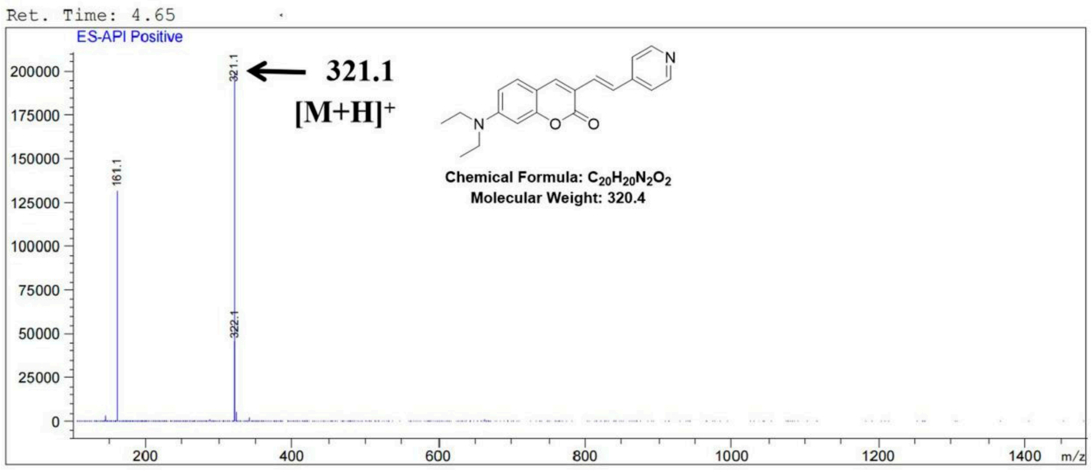

Figure S2. ESI-MS spectrum of **1** after reaction with Hg^{2+}.

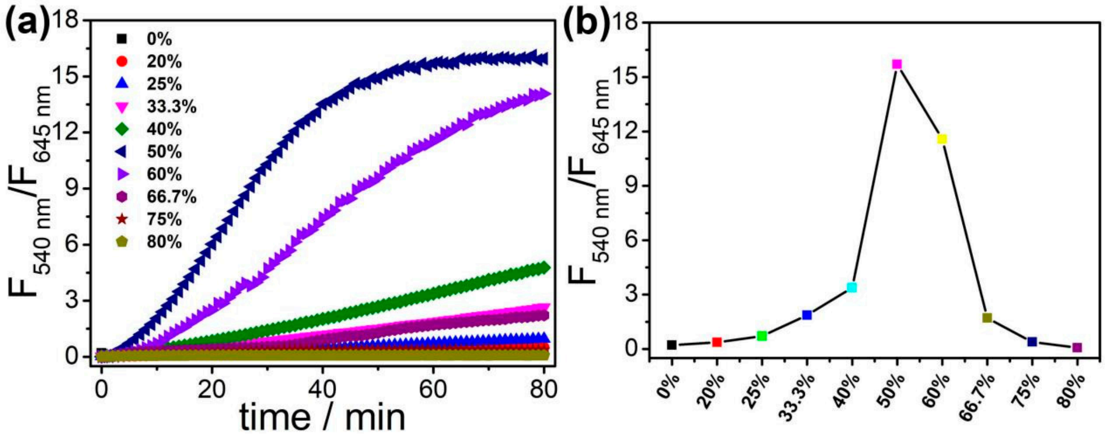

Figure S3. (a) The fluorescence intensity ratio ($F_{540\ nm}/F_{645\ nm}$) of **1** (5 μM) versus the reaction time after addition of Hg^{2+} (150 μM) in 10 mM PBS buffer solution with different ratios of DMSO (0-80%). (b) The relationship between the fluorescence intensity ratio ($F_{540\ nm}/F_{645\ nm}$) of **1** and ratios of DMSO (0-80%). Data were acquired at pH = 7.4, 25 °C, λ_{ex} = 500 nm.

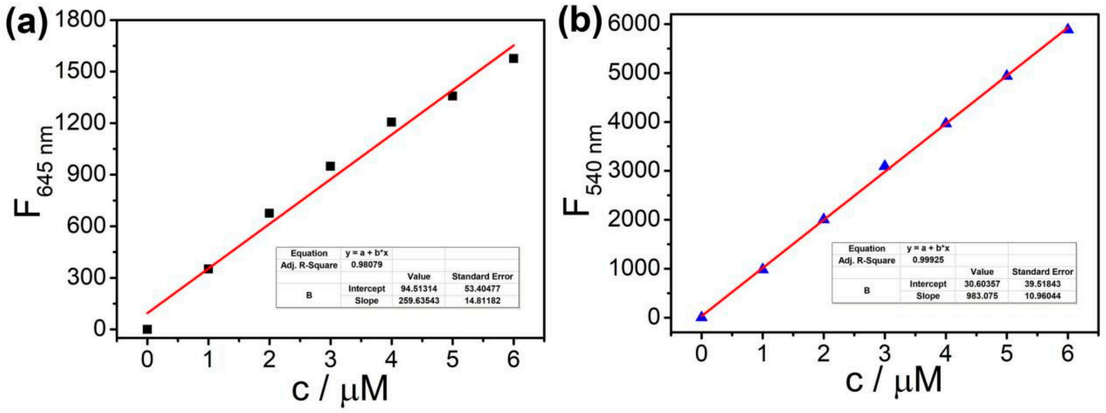

Figure S4. Plot of fluorescence intensity at 645 nm for **1** (black square) (a) and 540 nm for **2** (blue triangle) (b) against their concentrations in 10 mM PBS buffer solution/DMSO (1:1, v/v, pH = 7.4, 25 ºC, λ_{ex} = 500 nm).

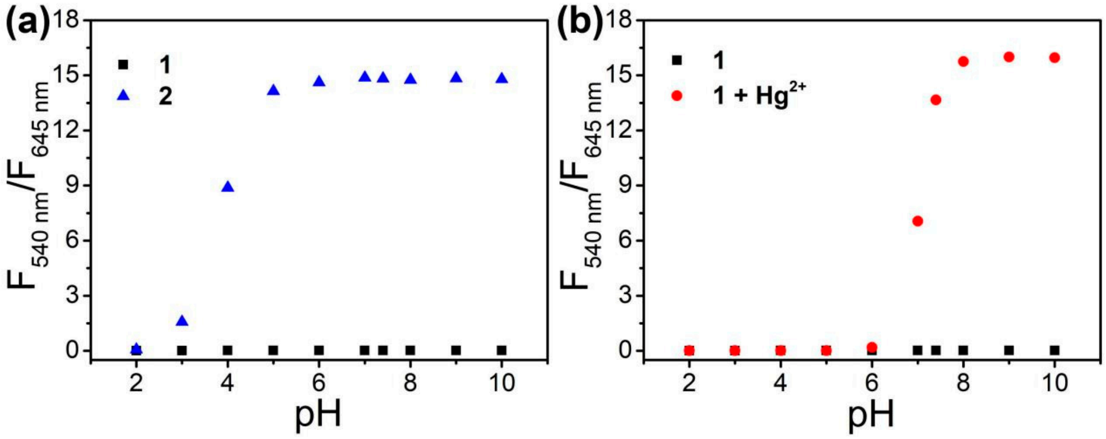

Figure S5. (a) Effect of pH on fluorescence intensity ratio of $F_{540\ nm}/F_{645\ nm}$ for **1** (5 μM) (black square) and **2** (5 μM) (blue triangle). (b) Effect of pH on fluorescence intensity ratio of $F_{540\ nm}/F_{645\ nm}$ for **1** (5 μM) in the absence (black square) and presence (red cycle) of Hg^{2+} (150 μM). Data were acquired in 10 mM PBS buffer solution/DMSO (1:1, v/v, pH = 7.4, 25 ºC, λ_{ex} = 500 nm).

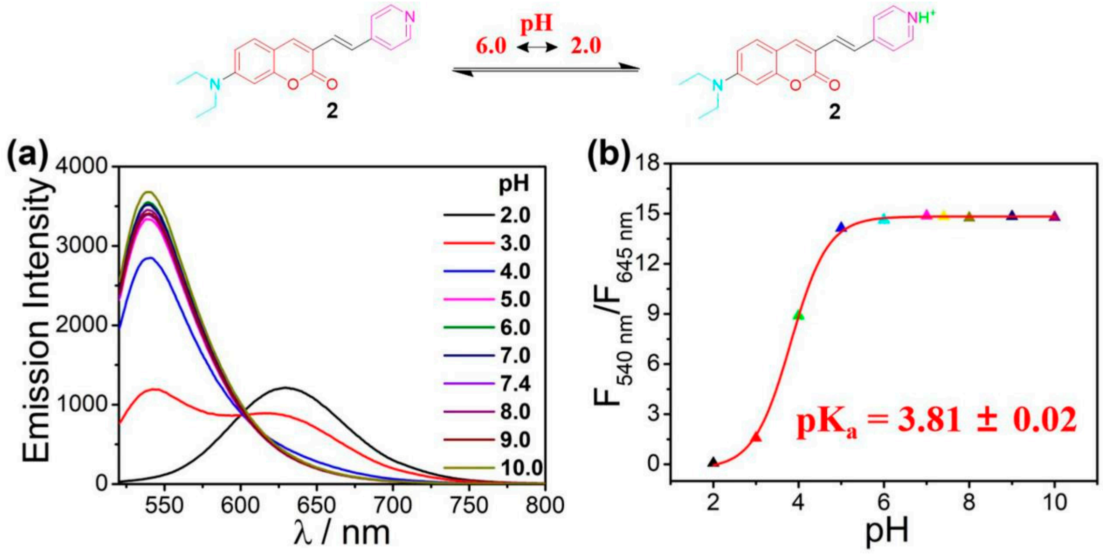

Figure S6. (a) Fluorescence spectra of **2** (5 μM) at various pH values in 10 mM PBS buffer solution/DMSO (1:1, v/v, pH = 7.4, 25 ºC, λ_{ex} = 500 nm). (b) The fluorescence intensity ratio ($F_{540\ nm}/F_{645\ nm}$) of **2** changes over the pH range of 2.0 - 10.0, the solid lines represent the non-linear least-squares fits to the experimental data.

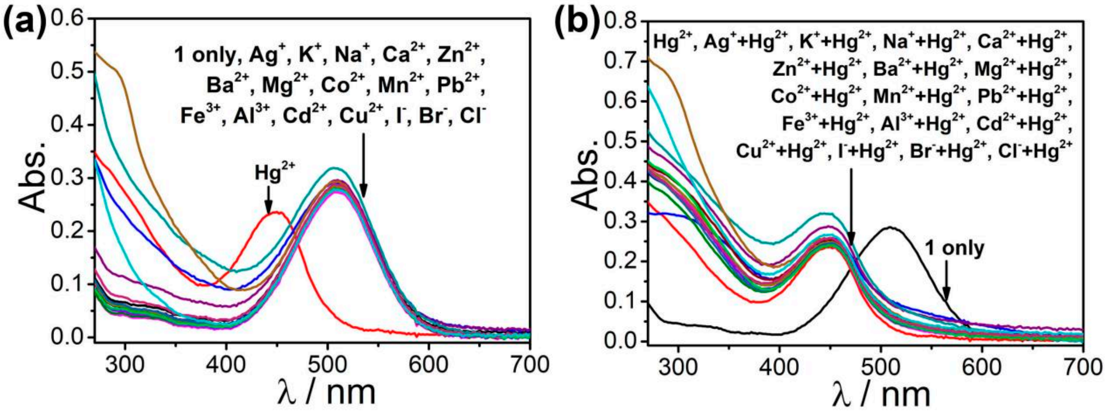

Figure S7. Absorption spectra of **1** (5 μM) after addition of various analytes (150 μM). (a) **1** and **1** treated with the marked analytes. (b) **1** treated with the marked analytes followed by Hg^{2+} and **1**. Data were acquired in 10 mM PBS buffer solution/DMSO (1:1, v/v, pH = 7.4, 25 ºC).

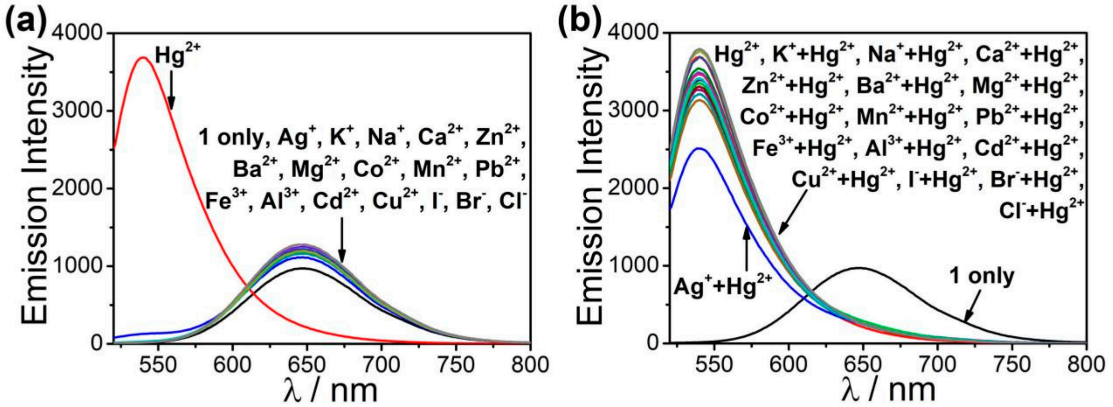

Figure S8. Fluorescence spectra of **1** (5 μM) after addition of various analytes (150 μM). (a) **1** and **1** treated with the marked analytes. (b) **1** treated with the marked analytes followed by Hg^{2+} and **1**. Data were acquired in 10 mM PBS buffer solution/DMSO (1:1, v/v, pH = 7.4, 25 ºC, λ_{ex} = 500 nm).

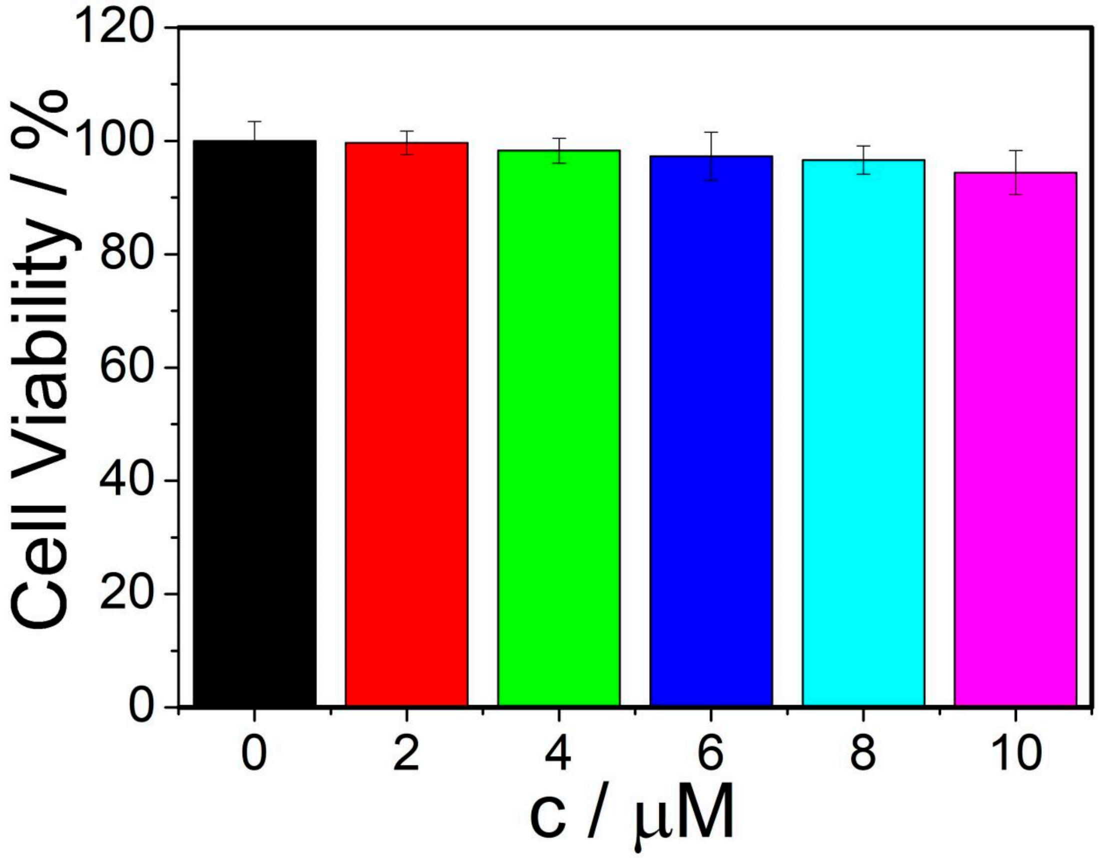

Figure S9. CCK-8 assay of HeLa cells in the presence of various concentrations of **1** (0, 2, 4, 6, 8, 10 μM) for 12 h at 37 °C.

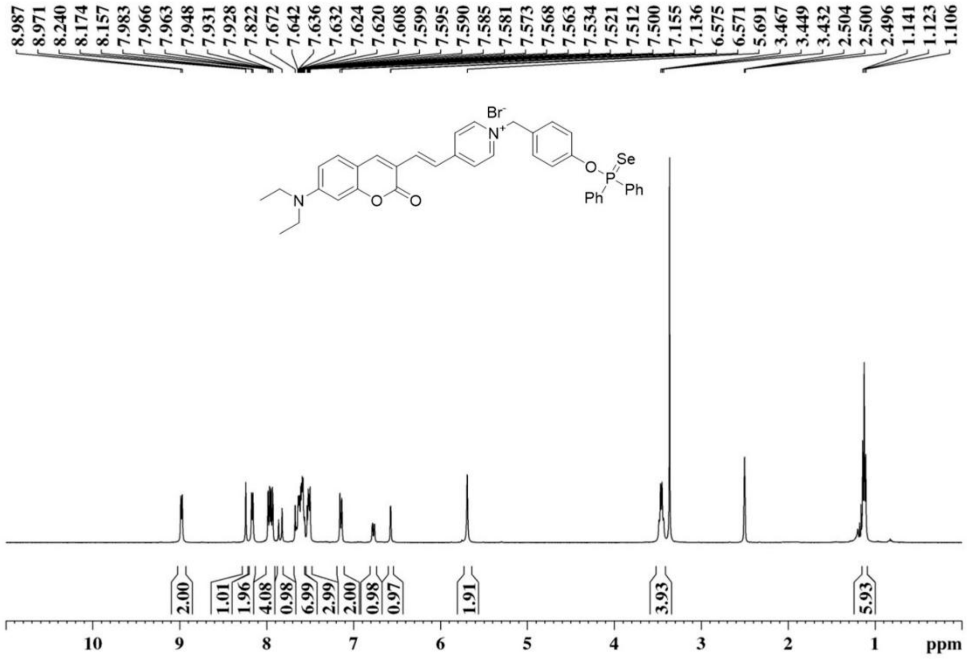

Figure S10. The ^{1}H NMR spectrum of **1** in DMSO-*d*$_6$.

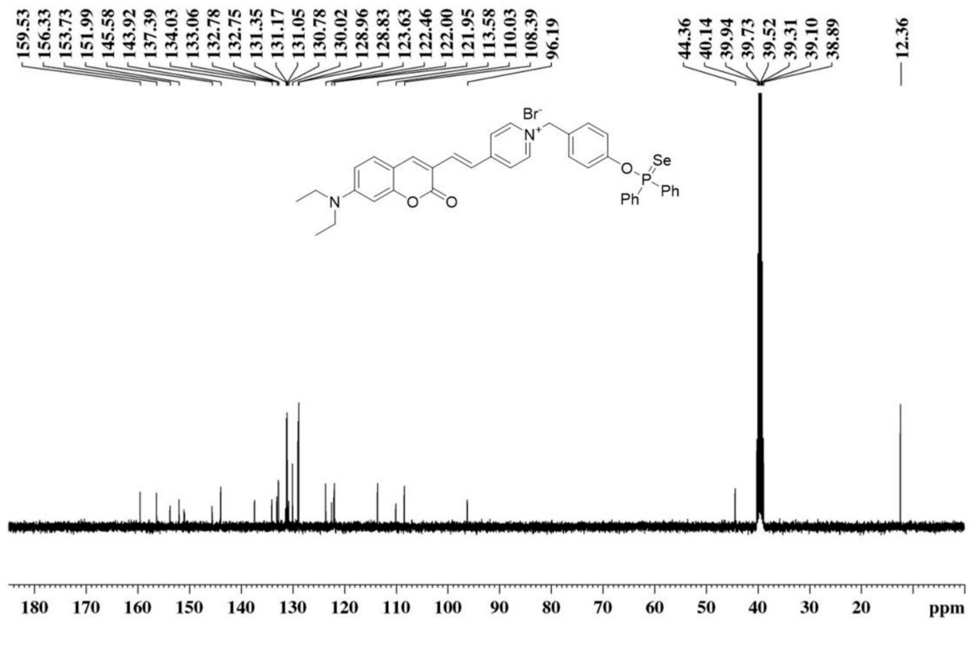

Figure S11. The ^{13}C NMR spectrum of **1** in DMSO-*d*$_6$.

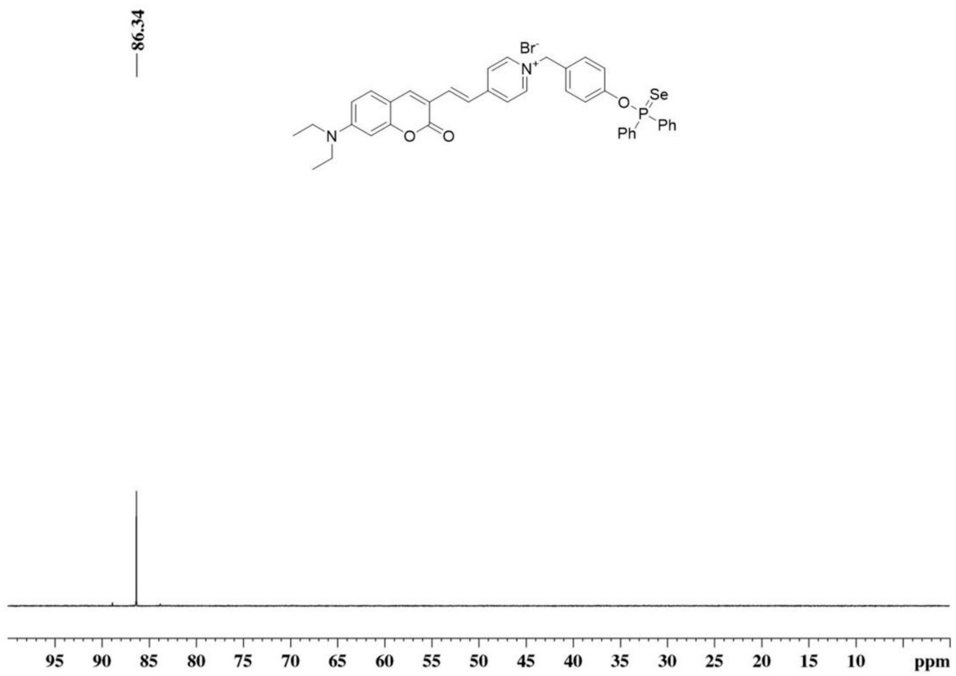

Figure S12. The ^{31}P NMR spectrum of **1** in DMSO-d_6.

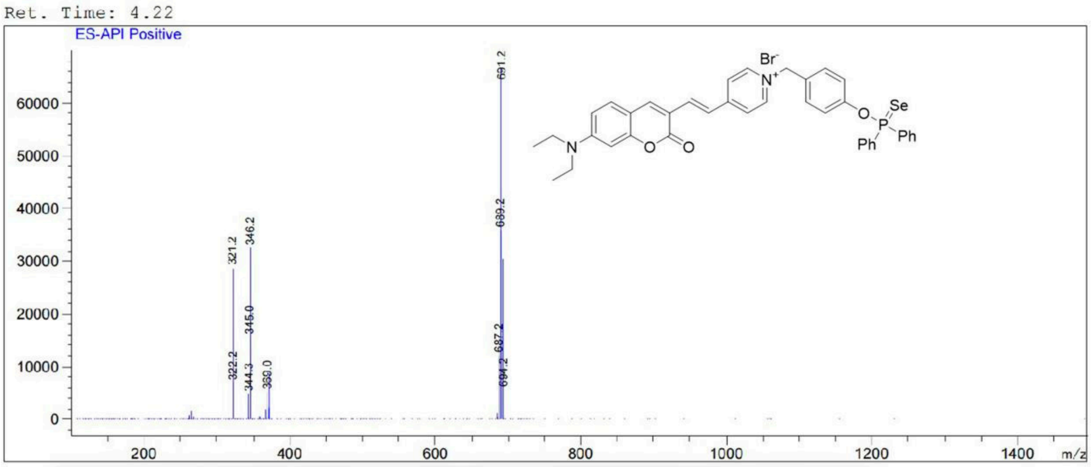

Figure S13. ESI-MS spectrum of **1**.
